# Temperature Characteristic Analysis of Electromagnetic Piezoelectric Hybrid Drive Motor

**DOI:** 10.3390/mi13060967

**Published:** 2022-06-18

**Authors:** Zheng Li, Xuetong Chen, Hui Zhao, Jinsong Wang, Shenhui Du, Xiaoqiang Guo, Hexu Sun

**Affiliations:** 1 School of Electrical Engineering, Yanshan University, Qinhuangdao 066004, China; gxq@ysu.edu.cn; 2 School of Electrical Engineering, Hebei University of Science and Technology, Shijiazhuang 050018, China; chenxuetong@stu.hebust.edu.cn (X.C.); zhaohuihbkd@163.com (H.Z.); wangjingsong@stu.hebust.edu.cn (J.W.); dushenhui@hebust.edu.cn (S.D.)

**Keywords:** electromagnetic drive, piezoelectric motor, loss, temperature rise, hybrid drive

## Abstract

Temperature rise has always been one of the main researchfocusesof the motor. When the temperature is too high, it will have a serious impact on the stability and reliability of motor performance. Due to the special structure of electromagnetic piezoelectric hybrid drive motor (EPHDM), the loss and temperature distribution of electromagnetic drive part and piezoelectric drive part werestudied. By analyzing the operation principle of the motor, the loss of each part wasresearched. On this basis, the loss of the electromagnetic driving part and piezoelectric driving part werecomputed by using the coupling iterative calculation method. The temperature contour map of the motor wasanalyzed by simulation, and the temperature characteristics of each part of the motor werestudied. Finally, the experimental verification of the prototype, the reliability of the theoretical model, and simulation results wereproved. The results showed that the temperature distribution of the motor is reasonable, the winding temperature is relatively high, and the core temperature and piezoelectric stator temperature are relatively low. The analytical and experimental methods are provided for the further study of heat source optimization.

## 1. Introduction

With the extensive research on the motor, the structure of the motors is more and more diverse, and the performance is also better and better. The EPHDMhas the preponderance of both electromagnetic and piezoelectric drives. It has the preponderances of high velocity, high thrust, high reliability, and long service life of the electromagnetic drive, as well as high dynamic response (millisecond level), high control accuracy, and passive self-locking of piezoelectric drive [1,2]. Therefore, hybrid drive motor has attracted extensive attention from scholars. However, due to the particularity of the structure of the hybrid drive motor, each link will bring more energy loss in the operation process. A considerable part of these losses is stored in the body with heat and transmitted according to its law, resulting in an increase in body temperature and affecting its working characteristics [3]. In the situation of a high-precision position servo, the change of motor characteristics will make it difficult to ensure accuracy. The loss of motor has attracted extensive attention. Reducing the loss can not only reduce the temperature rise and ensure accuracy but also improve the system stability and prolong the service life of the motor [4].

With the improving constantly and development of society, the field of engineering industry has strict requirements regarding the temperature of the motors [5]. Many professionals have researched the temperature characteristics of electromagnetic motors. Lingyu Gao et al. studied the relationship between temperature and motor performance. When the operating temperature changes from 20 °C to 180 °C, the permanent magnet remains non-demagnetized. At the same time, the relationship between temperature and performance is increased, providing a new direction for the motor R & D process [6,7]. Zezhi Xing et al. obtained a design scheme that meets the mechanical properties and temperature rise through multi-physical field analysis of the motor. The maximum temperature at the contact between the permanent magnet and the sheath reached 78.455 °C, which was lower than the limit temperature of the permanent magnet [8,9]. Xinghe Fu et al. calculated the steady-state temperature field of PMIHESG by using the three-dimensional FEM. By measuring the temperature, the predicted rotor eddy current loss is verified, and it is concluded that the rotor eddy current is on the side of the rotor salient near the molten pool [10,11]. These studies show that the electromagnetic drive part has a great impact on the loss of hybrid drive motors. The analysis of the loss and temperature characteristics of the electromagnetic drive is of great value to understanding the internal loss and further improving the performance of the motor.

At the same time, people have accumulated rich experience in the research of piezoelectric ultrasonic motors in theory and practice, and many scholars have also studied their temperature characteristics. Zheng Li et al. studied the temperature rise characteristics of the ultrasonic motors by establishing transient temperature field simulation. The results show that the temperature rise curve increases exponentially with time at the beginning, and then keeps stable with time as the motor enters the thermal temperature state [3]. Based on the fact that the performance of the ultrasonic motor is decided by the tribological characteristics of the contact interface, and the surrounding environment has a great impact on its characteristics, Xiaoliang Liu and others systematically researched the effect of temperature on the energy conversion of the motor with new friction materials for the first time. The results show that the long-time operation under a vacuum will cause arise in temperature and a reduction in friction, which will adversely affect the characteristics of TRUM-60. The output torque decreases sharply with the increase in temperature [12]. Based on the power loss theoretical model of ultrasonic motor, Xiaolong Lu et al. established a research method to evaluate the network loss of power. The results show that the friction loss accounts for the largest part of the medium loss, and analyzed the temperature distribution of the motor, which is highly consistent with the experimental results [13,14,15,16,17]. These studies show that the piezoelectric part also has a great impact on the loss of hybrid drive motors. The study of the loss and temperature characteristics of the piezoelectric drive is of great value to understanding the internal loss and further improving the performance of the motor.

In this paper, the loss and temperature characteristics of the EPHDM are studied. According to the particularity of the motor structure, the electromagnetic driving loss and piezoelectric driving loss are studied respectively, and the losses of each part are calculated. The motor temperature contour map is calculated through finite element simulation. Finally, the temperature measurement experimental platform is established with the micro temperature sensor as the core to checking the theoretical arithmetic and simulation outcome. The research results provide the theoretical and experimental basis for improving motor performance, improving motor operation stability, and reducing body temperature rise.

## 2. Structure and Driving Principle of Motor

### 2.1. Structure

**Figure 1:**
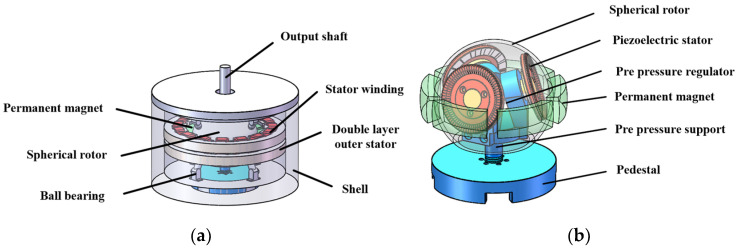
Structure of motor: (**a**) External electromagnetic drive; (**b**) Internal piezoelectric drive.

The structure of the EPHDM is shown in Figure 1, which is divided into two parts. Figure 1ais the external electromagnetic drive part and Figure 1b is the internal piezoelectric drive part. The external electromagnetic part is composed of a motor shell, double-layer external stator, stator winding, permanent magnet, spherical rotor, and ball bearing. The internal piezoelectric part is composed of a base, support shaft, preloading rod, three internal piezoelectric stators, and a spherical rotor [18]. The spherical rotor is a common structure of electromagnetic drive and piezoelectric drive.

**Figure 2:**
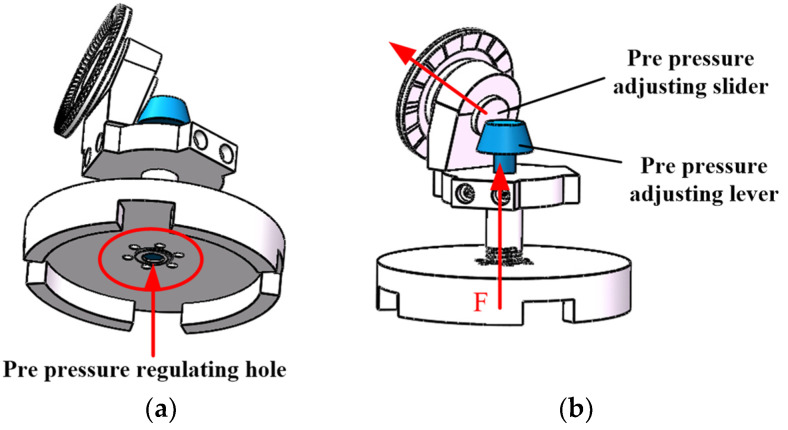
Pre pressure regulating system: (**a**) Bottom of the base; (**b**) Inside of the motor.

The double-layer stator of the electromagnetic drive part surrounds the equator of the rotor. The permanent magnet table is posted on the outside of the spherical rotor, six permanent magnets surround the equator of the rotor, and three N poles and three S poles are alternately distributed [2]. The three piezoelectric stators of the piezoelectric driving part are uniformly distributed in the spherical rotor, and the piezoelectric ceramic sheet (PCS) is attached to the bottom of the piezoelectric stator after silver plating polarization. The pre-pressure regulating device is shown in Figure 2, adjust the preload through the preload adjusting hole at the bottom of the base to make the preloading rod rise. At the same time, deduce the stator slider to make the stator rise and contact the rotor [19]. There are friction materials on the inner of the rotor, which can increase the driving force of the rotor and reduce the friction loss of the stator and rotor [3]. Ball bearings are used to support the rotor to decrease the gravity of the rotor itself and reduce the loss of the motor [20].

### 2.2. Driving Principle

#### 2.2.1. Electromagnetic Driving Principle

The operation principle of the electromagnetic drive part is in the light of the same-sex repulsion and opposite-sex attraction between magnetic poles [20]. By controlling the energization mode and size of the winding, the double-layer stator produces electromagnetic forces in different directions and sizes. Using same-sex repulsion and opposite-sex attraction between magnetic poles, the electromagnetic force will drive the rotor to produce rotational motion.

**Figure 3:**
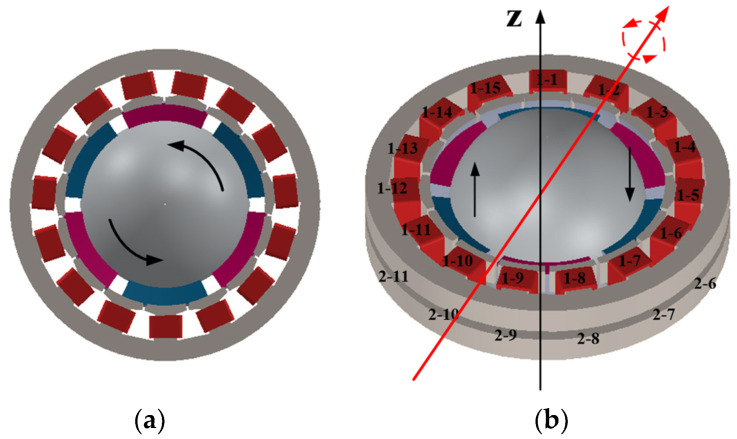
Electromagnetic driving principle: (**a**) Forward rotation; (**b**) Deflection.

When the same current brings to bear on the double-layer stator winding, the tangential electromagnetic force acting on the rotor will drive the motor to rotate around the *z*-axis, as shown in Figure 3a. When different currents are applied to the double-layer stator winding, the deflection movement of the motor can be realized by controlling the number of coils. As shown in Figure 3b, control winding coils 1-1, 1-2, 1-3, 2-14, 2-15, 2-6, 2-7, 2-8, 1-9, and 1-10 apply currents of the same size and direction to generate N-pole magnetic field, and control winding coils 2-1, 2-2, 2-3, 1-14, 1-15, 1-6, 1-7, 1-8, 2-9, and 2-10 apply currents of the same size and direction to generate S-pole magnetic field. The resultant electromagnetic force will drive the central axis of the motor rotor to deviate to the right. When energized in the reverse direction, the resultant electromagnetic force will drive the central shaft of the motor rotor to the left. Realize the deflection movement of the motor.

#### 2.2.2. Piezoelectric Driving Principle

**Figure 4:**
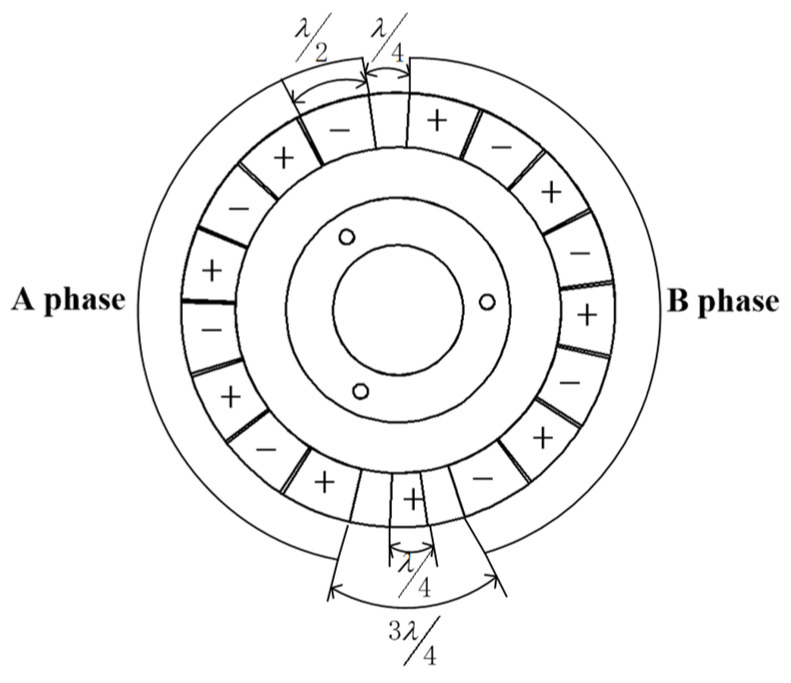
Polarization model of piezoelectric.

The operation principle of the piezoelectric driving part is in light of the inverse piezoelectric effect of piezoelectric ceramics. The polarization distribution of piezoelectric ceramics is shown in Figure 4. Apply the same sinusoidal and cosine AC voltage to two-phase PCSs A and B at the same time. Because the phase difference of PCSs arranged in space is 90°, the superposition of standing waves generated by two-phase voltage through the inverse piezoelectric effect of piezoelectric ceramics will form traveling waves. The high-frequency excitation makes the traveling wave pass through the amplified teeth of the stator matrix to produce a traveling wave with greater displacement [21]. The traveling wave on each piezoelectric stator will rotate around the stator axis. The rotation direction of the rotor can be controlled by controlling the direction of excitation applied by the three piezoelectric stators. The motion superposition of three piezoelectric stators will drive the rotor to produce forward rotation and deflection motion.

When the three piezoelectric internal stators are excited by the same sine, the directions of the traveling waves on the three stators are consistent, and the motor will realize the rotational movement around the *z*-axis. By controlling the three piezoelectric inner stators to excite sinusoidally in different directions, the orientation movement of the motor is realized when the orientation of the traveling waves on the three stators is positive and negative.

Because the EPHDM adopts a special stator and rotor structure, when the motor rotor completes the orientation movement, it can only complete the deflection movement within a certain angle range due to the limitation of the mechanical structure.

## 3. Loss Calculation

The loss of motor is not only the source of motor temperature rise but also an important factor to determine the efficiency of the motor. There are many losses. The loss of EPHDM is divided into electromagnetic loss and piezoelectric loss.

### 3.1. Electromagnetic Loss Calculation

**Figure 5:**
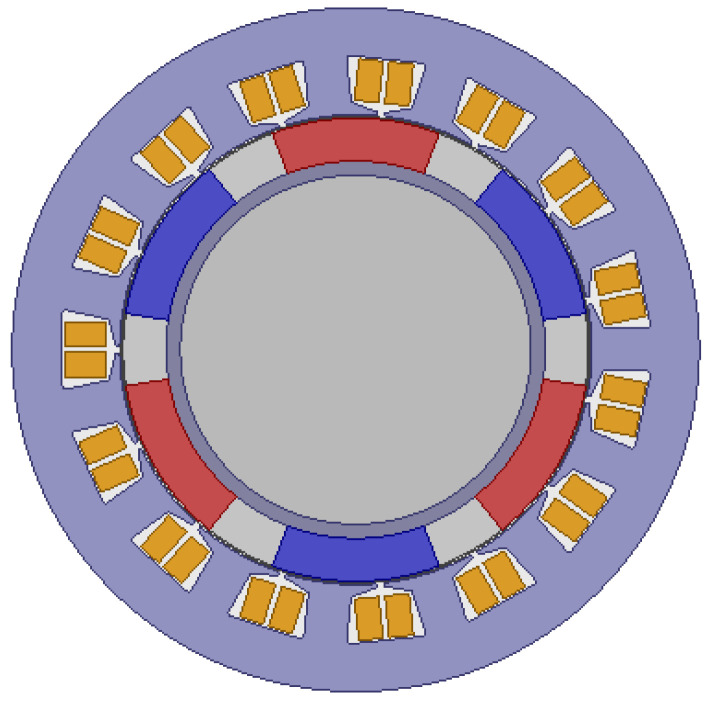
Two-dimensional models of the motor.

Electromagnetic loss includes stator core loss, winding copper loss, permanent magnet loss, rotor eddy current loss, and mechanical loss. Based on theoretical calculation and Maxwell simulation, the electromagnetic loss is analyzed. The two-dimensional model of the motor is established, as shown in Figure 5.

After the motor model is established, set the material parameters of each part. Use materials from Maxwell material library. The stator core adopts silicon steel sheet DW310-35, the stator winding adopts copper, the permanent magnet adopts neodymium iron boron Ndfe35, and the spherical rotor adopts aluminum. The relative permeability of copper is 0.999991, that of Ndfe35 is 1.0997785, and that of aluminum is 1.000021.

#### 3.1.1. Loss of Stator Core

There are three types of core loss. The first is the hysteresis loss, which is caused by the change of which is alternating magnetization or rotating magnetization in the motor. The second is eddy current loss, which is the induced current caused by the change of magnetic field, resulting in eddy current loss of iron core. The third is the additional loss, which is caused by the uneven distribution of eddy current and the fluctuation of magnetic flux density.

At present, the more recognized iron loss calculation model is Bertotti’s iron loss calculation. The calculation formula is as follows [22]:

(1) $$W_{Fe}=W_{h}+W_{c}+W_{e}$$

According to Bertotti’s iron loss separation theory, hysteresis loss $W_{h}$ is [23]:

(2) $$W_{h}=K_{h}f_{1}B_{m}^{a}$$

where, $K_{h}$ is the hysteresis loss coefficient, $f_{1}=50\mathrm{Hz}$ is AC frequency, and $B_{m}$ is the magnetic density amplitude.

The classical eddy current loss $W_{c}$ is [23]:

(3) $$W_{c}=\frac{\sigma \delta^{2}}{12\rho }\frac{1}{T}{\int_{0}^{T}\left(\frac{dB\left(t\right)}{dt}\right)}^{2}dt$$

where, σ is the conductivity of silicon steel sheet, δ is the thickness of silicon steel sheet, and ρ is the density of silicon steel sheet [24].

The magnetic density is decomposed into radial component and tangential component, and the calculation formula is transformed into:

(4) $$W_{c}=\frac{\sigma \delta^{2}}{12\rho }\frac{1}{T}\int_{0}^{T}\left[{\left(\frac{dB_{x}\left(t\right)}{dt}\right)}^{2}+{\left(\frac{dB_{y}\left(t\right)}{dt}\right)}^{2}\right]dt$$

Additional loss $W_{e}$ is [22]:

(5) $$W_{e}=\frac{\sqrt{\sigma GV_{0}S}}{\rho }\frac{1}{T}{\int_{0}^{T}\left|\frac{dB\left(t\right)}{dt}\right|}^{1.5}dt$$

where, G and $V_{0}$ are the correlation coefficient of magnetic properties of silicon steel sheet, and S are the surface cross-sectional area of silicon steel sheet.

The magnetic density is decomposed into radial component and tangential component, and the calculation formula is transformed into:

(6) $$W_{e}=\frac{\sqrt{\sigma GV_{0}S}}{\rho }\frac{1}{T}\int_{0}^{T}\left[{\left|\frac{dB_{x}\left(t\right)}{dt}\right|}^{1.5}+{\left|\frac{dB_{y}\left(t\right)}{dt}\right|}^{1.5}\right]dt$$

When the magnetic density waveform is sinusoidal, that is, $B\left(t\right)=B_{m}\sin\left(2\pi f_{1}t\right)$, the iron loss is [23]:

(7) $$W_{h}=K_{h}f_{1}B_{m}^{a}+K_{c}f_{1}{}^{2}B_{m}^{2}+K_{e}f_{1}{}^{1.5}B_{m}^{1.5}$$

where, $K_{c}$ is eddy’s current loss coefficient, and $K_{e}$ is the additional loss coefficient.

**Figure 6:**
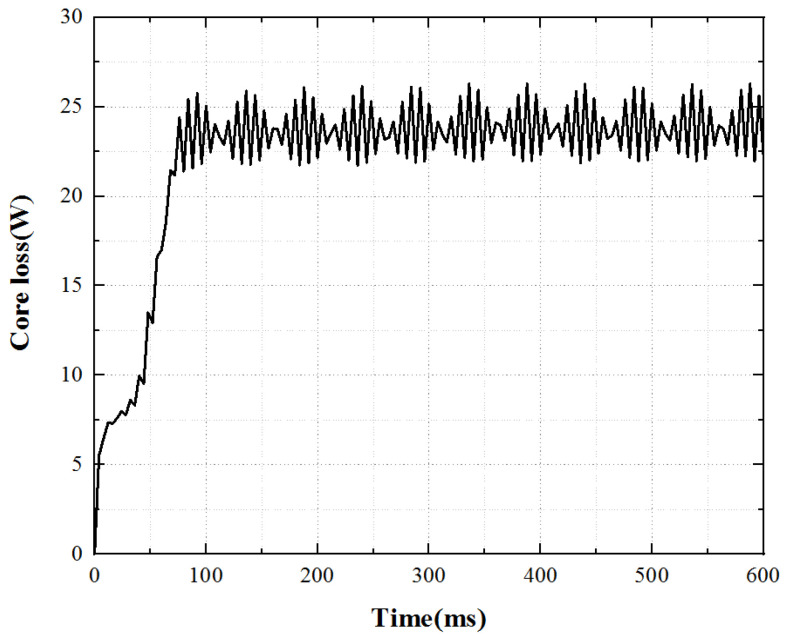
Core loss curve.

Under the working state of the open-loop constant voltage drive of the motor, the core loss is calculated by using Maxwell software to analyze the stator core loss, and the core loss curve as shown in Figure 6 is obtained. The core loss has a sudden change process when the motor is just started, and then gradually runs smoothly. The average stator core loss underrated working conditions can reach 23.8 W.

#### 3.1.2. Copper Consumption of Stator Winding

The copper loss of winding is due to the existence of conductor resistance. After adding a three-phase AC source, it will produce energy loss. Because the motor speed is low, the multiple harmonics in the current input by the frequency converter have little impact on the motor loss, and the skin effect caused by uneven current distribution can also be ignored.

The calculation formula of copper consumption $W_{Cu}$ can be expressed as [23]:

(8) $$W_{Cu}=mI^{2}R$$

where, m is the number of winding phases, I is the effective value of current, and R is the resistance value of winding.

When the motor starts to operate at normal temperature, the temperature of the body will ascend owing to loss, and the temperature of the stator winding will rise due to copper consumption. The resistivity of copper material will increase with the rise of motor temperature, which will increase the phase resistance of the motor and its impact on copper consumption.

The calculation formula of conductor resistance value varying with temperature is [23]:

(9) $$R=\rho \frac{l}{S}=\rho_{0}\left[1+\partial_{0}\left(t-t_{0}\right)\right]\frac{l}{S}$$

where, ρ is the winding conductor resistivity, l is the conductor length, S is the conductor cross-sectional area, $\rho_{0}$ is the resistivity at the reference temperature, and $\partial_{0}$ is the temperature coefficient of the conductor material.

The calculation shows that the copper consumption of stator winding is 31.1 W under the rated working condition of the motor.

#### 3.1.3. Eddy Current Loss of Permanent Magnet

In general, it is considered that the speed of the stator’s fundamental magnetic potential is the same as that of the rotor. In principle, the magnetic field of a permanent magnet remains unchanged. However, there will be errors in the actual manufacturing process and materials. When the motor rotates, the permeability in the magnetic circuit will change, and the magnetic field passing through the permanent magnet will change accordingly. In addition, there are high-order harmonics in the motor different from the velocity of the rotor permanent magnet, in which current will be induced and eddy current loss will be formed.

The axial density of the eddy current of a permanent magnet can be expressed as [25]:

(10) $$J_{z}=\nabla \times \left[\frac{1}{\mu_{p}}\left(\nabla \times A_{z}-B_{p}\right)\right]+\sigma_{p}\frac{\partial A_{z}}{\partial t}$$

where, $\mu_{p}$ is the permeability of the permanent magnet, $A_{z}$ is the axial vector magnetic density, $B_{p}$ is the residual magnetism of the permanent magnet, and $\sigma_{p}$ is the conductivity of the permanent magnet.

Eddy’s current loss density is [25]:

(11) $$W_{pw}=L_{p}\underset{S_{p}}{∯}\frac{{\left|J_{z}\right|}^{2}}{\sigma_{p}}dS_{p}$$

where, $S_{p}$ is the cross-sectional area of the permanent magnet, and $L_{p}$ is the axial length of the permanent magnet.

**Figure 7:**
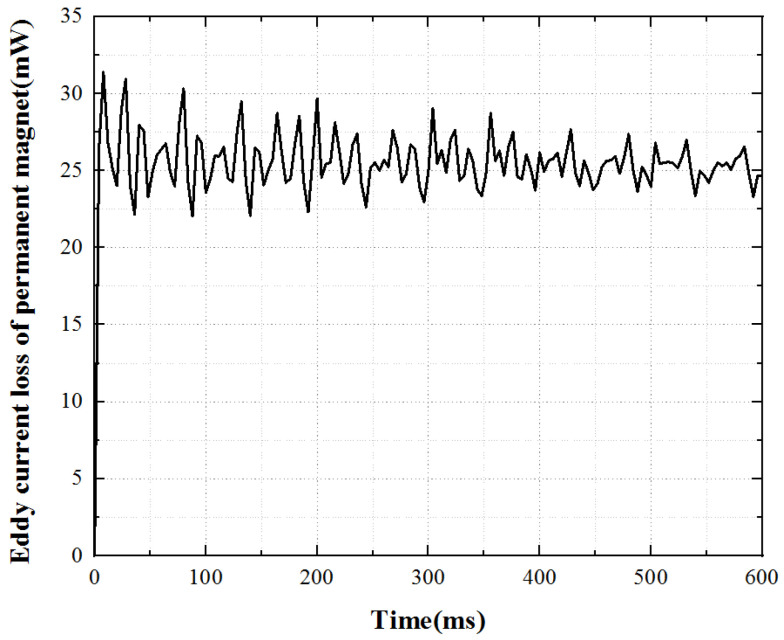
Eddy current loss curve of the permanent magnet.

Under the working state of the open-loop constant voltage drive of the motor, the eddy current loss in the permanent magnet can be calculated by using Maxwell software settings, and the waveform curve of eddy current loss in the permanent magnet can be obtained, as shown in Figure 7. The eddy current loss of permanent magnet underrated working conditions is very small, but the loss fluctuates greatly. The average loss of underrated working conditions is 0.026 W.

#### 3.1.4. Rotor Eddy Current Loss

**Figure 8:**
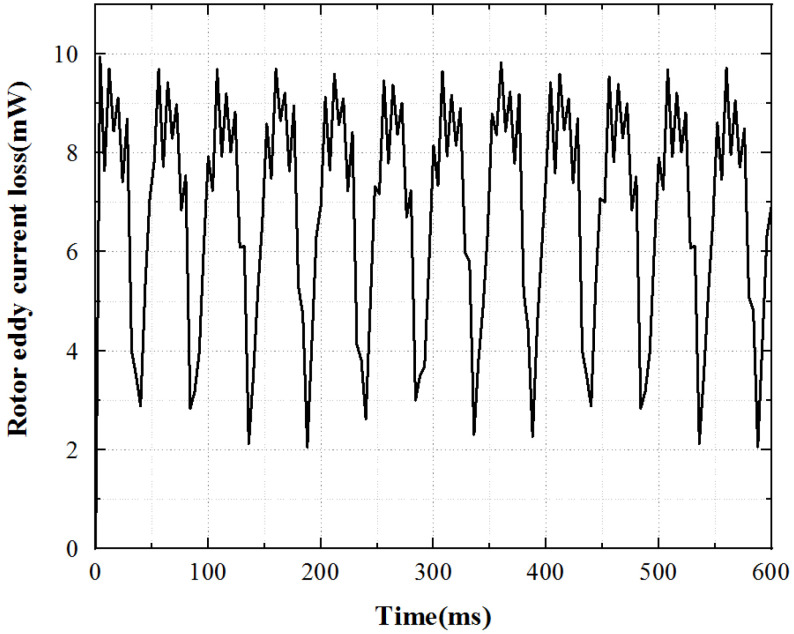
Rotor eddy current loss curve.

The eddy current loss of the motor rotor is very small. The loss of underrated working conditions is obtained through Maxwell simulation, as shown in Figure 8. The loss fluctuates greatly, and the average loss is 0.0073 W.

#### 3.1.5. Mechanical Loss

The mechanical loss in the motor is affected by the motor’s motion mechanism and structural material parameters, which generally include two aspects: one is the ventilation loss affected by the motor speed, structure, and the fluid properties in contact with the motor; One is the bearing loss affected by processing technology.

Ventilation loss is [25]:

(12) $$W_{f}=C_{f}\rho_{0}\pi n^{3}r_{r}^{4}L_{r}$$

where, $C_{f}$ is the friction coefficient, $\rho_{0}$ is the density of the surrounding medium, n is the motor speed, $r_{r}$ is the radius of the rotor, and is $L_{r}$ is the effective length of the rotor.

The friction coefficient $C_{f}$ is [25]:

(13) Cf=0.0152Reδ0.241+8724ReaRea20.38Reδ=ρ0nrrδμ1Rea=ρ0νa2δμ1

where, Reδ is the radial Reynolds coefficient, Rea is the tangential Reynolds coefficient, $\mu_{1}$ is relative permeability, and $\nu_{a}$ is medium viscosity coefficient.

Bearing loss is [23]:

(14) $$W_{b}=C_{b}D_{m}^{3}\omega_{r}$$

where, $C_{b}$ is the bearing coefficient, and $D_{m}$ is the bearing diameter.

Because the mechanical loss is very small in small, and medium-sized motors, it is generally 1% to 2% of the total power. Therefore, this factor is generally not considered when studying the distribution of the motor temperature field.

To sum up, the loss values of each part of the electromagnetic drive in Table 1 are calculated.

According to references [25,26], the partial losses of traditional permanent magnet synchronous motors are shown in Table 2.

It can be seen from Table 1 and Table 2 that the loss of the EPHDM studied in this paper is greatly reduced due to the particularity of its structure. Because the inner part of the motor is a spherical rotor, and the outer stator is attached around the rotor equator, the effective area of the outer stator is reduced and the loss is reduced. As well, the motor rotor is made of aluminum, which eliminates the core loss of the rotor. It provides a reference for the follow-up study.

### 3.2. Piezoelectric Loss Calculation

Piezoelectric loss includes piezoelectric ceramic loss, stator deformation loss, contact interface friction loss, and so on. Based on theoretical calculation, the piezoelectric loss is analyzed.

#### 3.2.1. Vibration Loss of PCS

Dielectric loss includes dielectric loss and dielectric loss of ceramic structure.

Structural damping loss is [3]:

(15) $$W_{p1}=nf_{2}\int_{V_{piezo}}\delta W_{s1}dV$$

where, n=9 is the number of stator modes, $f_{2}$ is the excitation signal frequency. $\delta W_{s1}$ is internal friction loss.

The dielectric loss is [3]:

(16) $$W_{p2}=2\pi f_{2}\varepsilon \tan\delta \frac{U^{2}}{H_{p}}V_{piezo}$$

where, ε is the dielectric constant, tanδ is the dielectric loss coefficient, $U=U_{A}=U_{B}$ is the excitation voltage, $H_{p}$ is the thickness of the PCS, and $V_{piezo}$ is the volume of the PCS.

Therefore, the total loss $W_{vp}$ of piezoelectric ceramics is:

(17) $$W_{vp}=W_{p1}+W_{p2}$$

The calculation shows that the loss of piezoelectric ceramics is 11.9 W under the open-loop constant voltage driving state of the motor.

#### 3.2.2. Stator Deformation Loss

For the plate and shell continuum, when the continuum vibrates, the internal mechanical energy is transformed into thermal energy and dissipated.

According to Kelvin’s viscous damping theory, the internal stress of the structure δ is [3]:

(18) $$\delta =E\left(s+\chi \frac{\partial s}{\partial t}\right)$$

where, E is the elastic modulus, s is the strain vector, and χ is the viscous damping coefficient [3].

(19) $$\chi =\frac{2\eta }{\omega_{n}}$$

where, η is the damping ratio, and $\omega_{n}$ is the vibration angular frequency.

The change of internal energy caused by stress in each bending of the stator is expressed as [3]:

(20) $$\delta W_{in}=\frac{1}{2}\int E\left(s+\chi \frac{\partial s}{\partial t}\right)ds=\delta W_{k}+\delta W_{s1}$$

The energy loss caused by internal friction is [3]:

(21) $$\delta W_{s1}=\frac{1}{2}\int E\chi \frac{\partial s}{\partial t}ds=\frac{1}{2}{\int_{0}^{\frac{2\pi }{w_{n}}}\left(\frac{\partial s}{\partial t}\right)}^{T}E\chi \frac{\partial s}{\partial t}dt$$

Therefore, the structural damping loss $W_{s}$ of the whole stator is [3]:

(22) $$W_{s}=W_{ring}+W_{tooth}=nf_{2}\left(\int_{V_{ring}}\delta W_{s1}dV+\int_{V_{tooth}}\delta W_{s1}dV\right)$$

where, $W_{ring}$ and $W_{tooth}$ respectively represent the damping loss of stator matrix and stator teeth, and $V_{ring}$ and $V_{tooth}$ respectively represent the volume of stator matrix and stator teeth.

The calculation shows that the loss caused by stator deformation is 1.31 W under the open-loop constant voltage driving state of the motor.

#### 3.2.3. Friction Loss of Stator and Rotor

During the operation of the motor, the contact interface is affected by the force, which is disintegrated into tangential force and radial force. Among them, the tangential force drives the motor, but there is a certain resistance due to the friction drive, resulting in the circumferential loss of the motor [27]. The radial force causes the radial slide of the motor, hinders the normal movement of the motor, and causes the radial loss of the motor [28,29].

The circumferential loss of the contact surface within one wavelength can be calculated as [3]:

(23) $$W_{f_{\tau v}}=\iint f_{\tau }|V_{\tau \theta 0}\sin n\theta -v_{\tau }|dS$$

where, $f_{\tau }$ is the tangential force, $V_{\tau \theta 0}$ is the maximum tangential velocity of the traveling wave in the stator, $v_{\tau }$ is the tangential velocity of the rotor, and S is the contact area.

The radial loss of the contact surface within one wavelength can be calculated as [3]:

(24) $$W_{f_{\tau u}}=\iint f_{\tau }|V_{\tau r0}\cos n\theta |dS$$

where, $V_{\tau r0}$ is the maximum radial velocity of the traveling wave in the stator.

Therefore, the friction loss $W_{f}$ in the whole contact area is:

(25) $$W_{f}=n\left(W_{f_{\tau v}}+W_{f_{\tau u}}\right)$$

The calculation shows that the friction loss is 16.68W when the motor is driven by open-loop constant voltage.

To sum up, the loss values of each part of the piezoelectric drive in Table 3 are calculated.

According to reference [30], the loss of the traditional traveling wave-type rotating ultrasonic motor is shown in Table 4.

It can be seen from Table 3 and Table 4 that the loss of the piezoelectric part of the motor is slightly higher than that of the traditional ultrasonic motor. Because of the particularity of the motor structure, the motor loss increases while increasing the motor motion degrees of freedom and realizing the multi-degrees of freedom motion. In the follow-up, we will conduct in-depth research on reducing motor loss and optimizing the motor.

## 4. Temperature Field Analysis

The energy loss generated by the motor during operation causes the temperature to ascend in the body. The loss of the motor is mainly divided into two parts: electromagnetic loss and piezoelectric loss. Electromagnetic loss includes stator core loss and winding copper loss. Piezoelectric loss includes friction loss and piezoelectric ceramic loss. This paper uses ANSYS Workbench multi-physical field simulation to analyze the contour map and temperature rise characteristic curve of engine block temperature field caused by these four kinds of losses.

Material parameters are shown in Table 5. When analyzing the temperature field, set the ambient temperature and initial temperature to 25 °C, and the heat transfer coefficient of the motor under natural convection conditions is 12 W/m^2^K.

### 4.1. Electromagnetic Temperature Analysis

**Figure 9:**
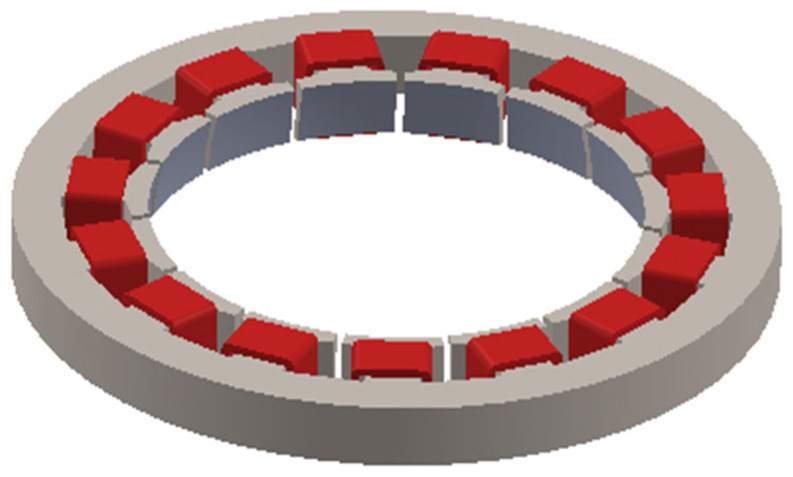
Outer stator model.

When simulating the temperature field of the electromagnetic part, because the double-layer stator is completely symmetrically distributed, the upper stator is selected as the research object, as shown in Figure 9.

#### 4.1.1. Analysis of Temperature of Stator Core under Independent Action

**Figure 10:**
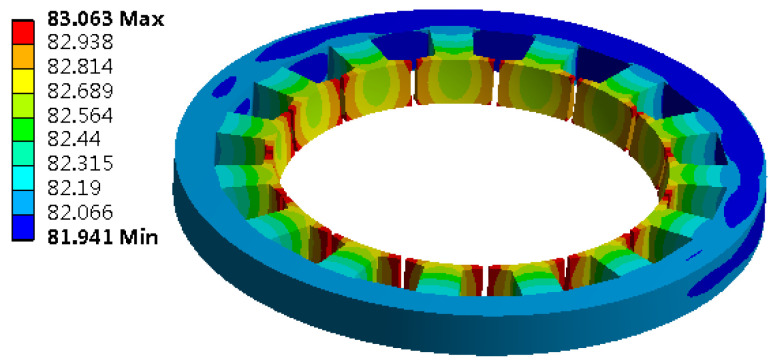
Contour diagram of stator core temperature.

The stator core is used as the heat source, which is added to the stator core unit in the form of calorific load per unit volume for the solution, and its temperature contour is obtained, as shown in Figure 10.

The calorific value per unit volume caused by stator core loss is [25]:

(26) qh=WhVh

where, $W_{h}$ is the loss of the stator core, and $V_{h}$ is the volume of the stator core.

**Figure 11:**
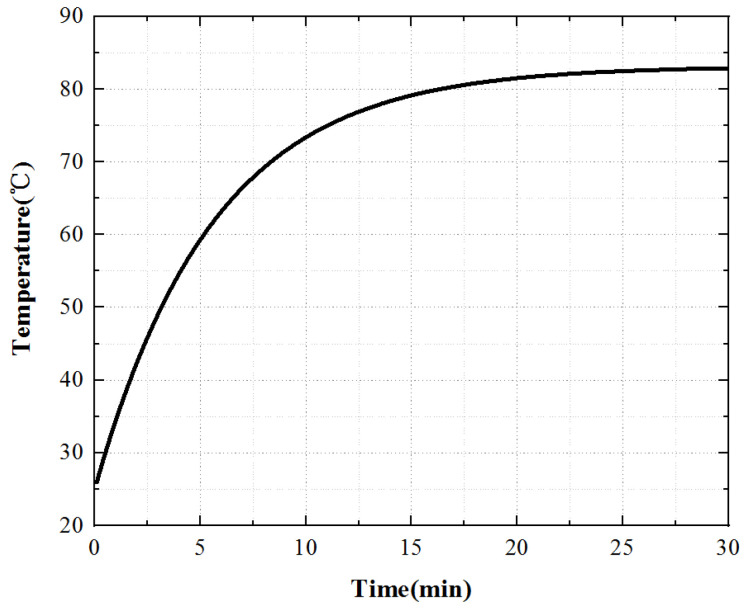
Temperature rise characteristic curve of the stator core.

When the stator core acts alone, the heat scatter is nonuniform, the temperature of the stator teeth is the highest, and the temperature of the stator yoke is low. Select a point on the stator tooth to obtain the temperature rise curve of this point, as shown in Figure 11.

For the stator core, its surface temperature first raises rapidly, then raises slowly, and finally enters the thermal stable state. At 0~10 min, the temperature increases rapidly, at 10~20 min, the temperature increases slowly, and the temperature remains constant after 20 min.

#### 4.1.2. Analysis of Temperature of the Stator Winding under Independent Action

**Figure 12:**
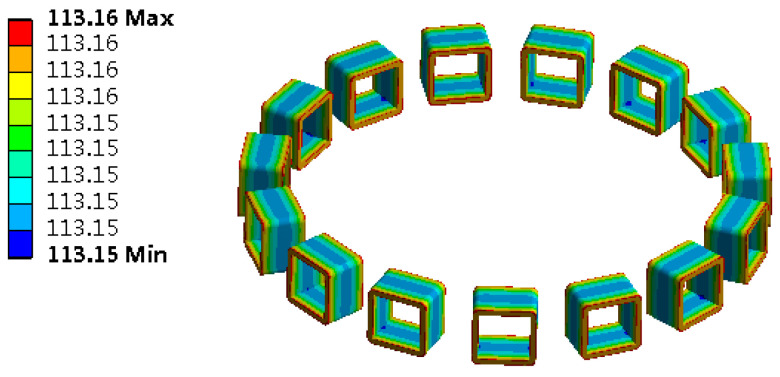
Contour diagram of stator winding temperature.

As the heat source, the stator winding establishes the transient heat conduction model of the winding, adds it to the stator winding unit in the form of a calorific load of the unit volume, and obtains the contour map of the temperature of the stator winding, as shown in Figure 12.

The calorific value per unit volume caused by stator winding loss is [25]:

(27) qCu=WCuVCu

where, $W_{Cu}$ is the loss of stator winding, and $V_{Cu}$ is the volume of the stator winding.

**Figure 13:**
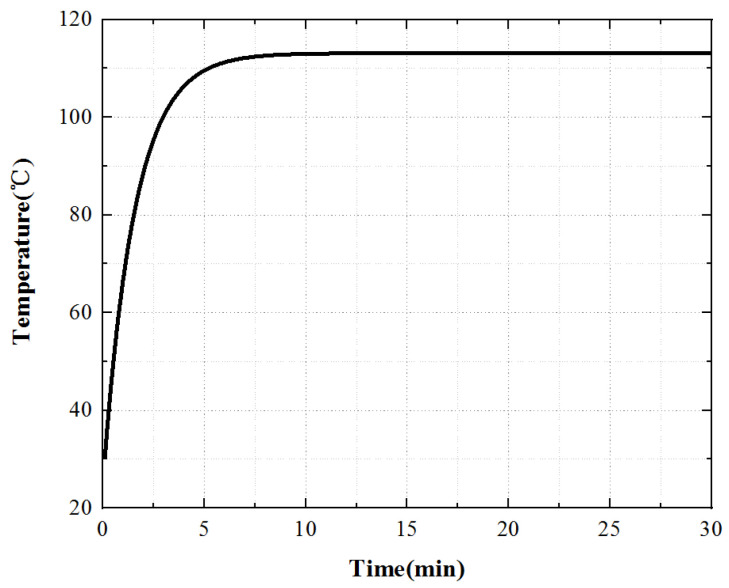
Temperature rise characteristic curve of the stator winding.

When the stator winding acts alone, the heat distribution of the winding is also uneven. The temperature at both ends of the winding is high and the temperature at the center of the winding is low, which is distributed in steps in the radial direction. Select a point on the winding surface to obtain the temperature rise curve of this point, as shown in Figure 13.

For the stator winding, the growth rate of winding surface temperature is first fast and then slow, which is consistent with the growing trend of iron core temperature rise, and finally, the motor enters the thermal stability state. At 0~3.5 min, the temperature increases rapidly. At 3.5~6 min, the temperature increases slowly. After 6 min, the temperature remains constant, and the motor enters a thermally stable state.

### 4.2. Piezoelectric Temperature Analysis

When simulating the temperature field of the piezoelectric part, one stator is selected for simulation analysis because the three piezoelectric inner stators are symmetrically distributed.

#### 4.2.1. Temperature Analysis of Piezoelectric Ceramics under Single Action

**Figure 14:**
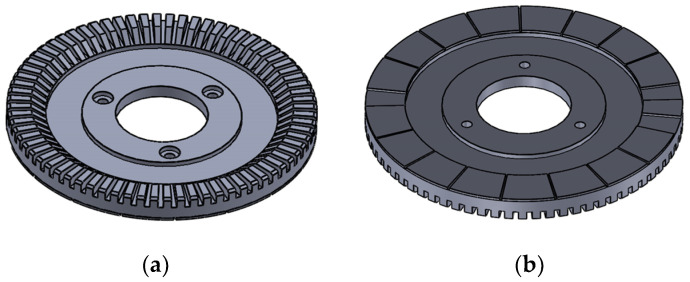
Piezoelectric stator model: (**a**) Stator front; (**b**) Stator back.

**Figure 15:**
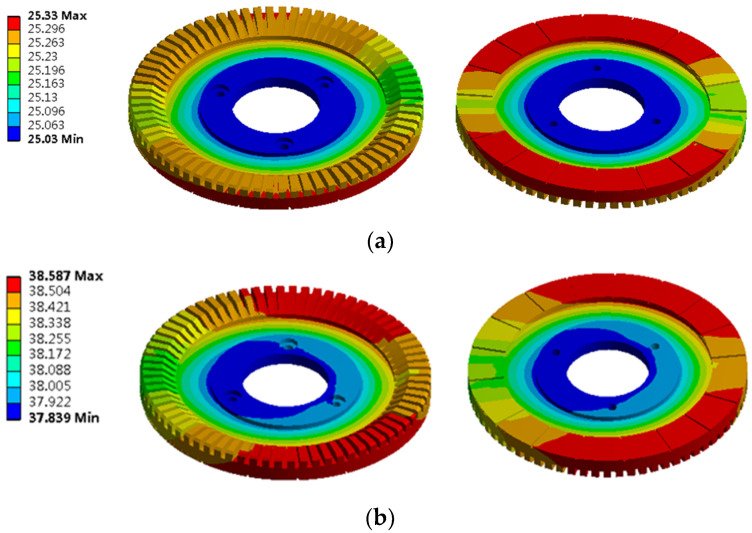
Contour map of piezoelectric stator temperature: (**a**) 20 s temperature contour map; (**b**) 30 min temperature contour map.

The PCS is used as the heat source, and the transient heat conduction model of the piezoelectric stator is shown in Figure 14. Add the load in the form of a calorific load of unit volume to the piezoelectric ceramic element and solve it to obtain its temperature contour map, as shown in Figure 15.

The calorific value per unit volume caused by the loss of PCS is [3]:

(28) qvp=WPVpiezo

where, $W_{P}$ is the loss of stator core, and $V_{piezo}$ is the volume of the PCS.

**Figure 16:**
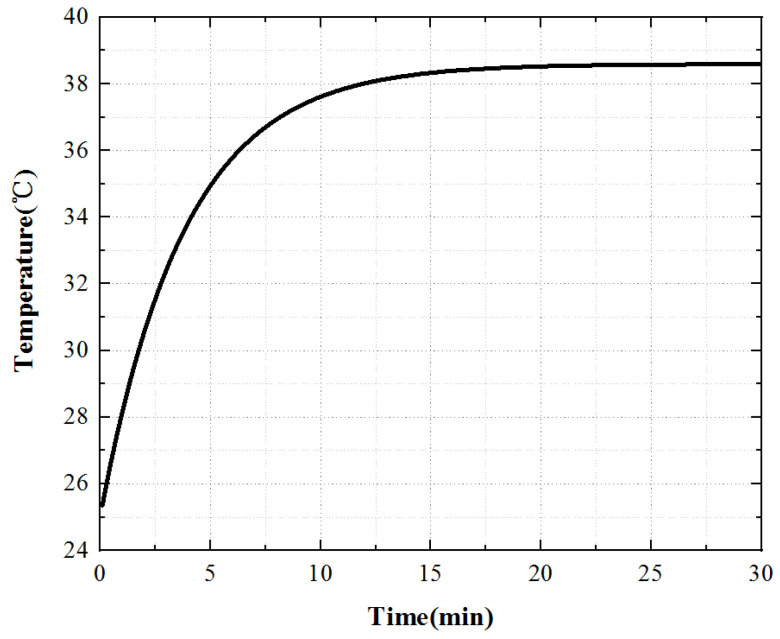
Temperature rise characteristic curve of the piezoelectric stator.

Under the action of the PCS alone, the temperature of the edge cylindrical part passedwith the PCS is the highest. Because the PCS passedto the bottom of the piezoelectric stator matrix, the temperature of the bottom of the stator is the highest. With the increase in time, the temperature increases gradually, and the heat energy is delivered to the upper of the stator substrate. After working for 30 min, the temperature of the upper surface of the stator substrate is equal to that of the PCS. Select a point of the stator tooth to obtain the temperature rise curve of this point, as shown in Figure 16.

From the temperature rise curve in Figure 16, we can know that when the piezoelectric ceramic acts alone, the growth trend of its surface temperature is consistent with that of the iron core temperature, which increases rapidly at first, then slowly, and finally enters the thermal stable state. However, the temperature rise time is slightly different from that of the iron core. The temperature increases rapidly at 0~8 min, slowly at 8~15 min, and when t > 15 min, the temperature tends to be stable and the motor enters a thermally stable state.

#### 4.2.2. Temperature Analysis of Friction Heat Generation Alone

**Figure 17:**
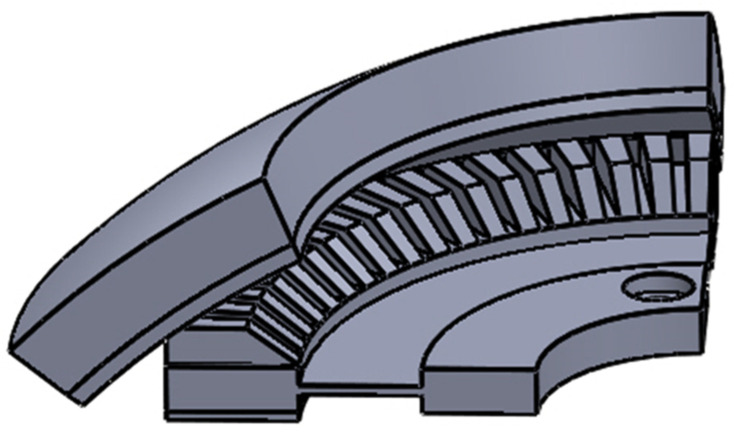
Friction contact model.

During the temperature field simulation, due to the symmetry of the motor, select 1/4 of a stator and rotor model to establish a transient heat conduction model, as shown in Figure 17.

The heat generation rate of the friction interface is:

(29) qm=WmSm

where, $W_{m}$ is the friction loss, and $S_{m}$ is the area of the friction interface.

**Figure 18:**
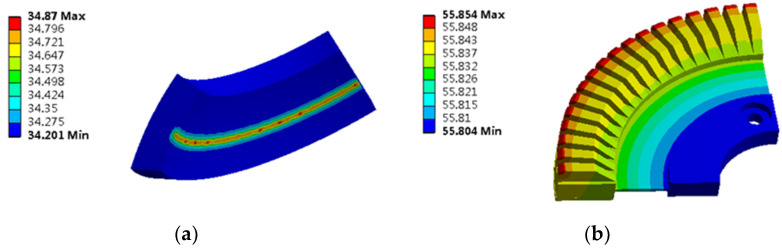
Contour map of contact interface temperature: (**a**) Contour map of rotor temperature; (**b**) Contour map of stator temperature.

During the simulation analysis, the heat flux is used as the load to act on the friction material sheet of the contact interface, and the solution is carried out to obtain the temperature contour map of the stator and rotor, as shown in Figure 18.

Under the action of friction heat generation alone, the surface temperature of the rotor is lower than that of the stator teeth. The main reason is the existence of friction materials on the inner, which effectively inhibits the transfer of heat to the rotor surface. From the point of view of temperature analysis, the selection of friction materials is very important.

**Figure 19:**
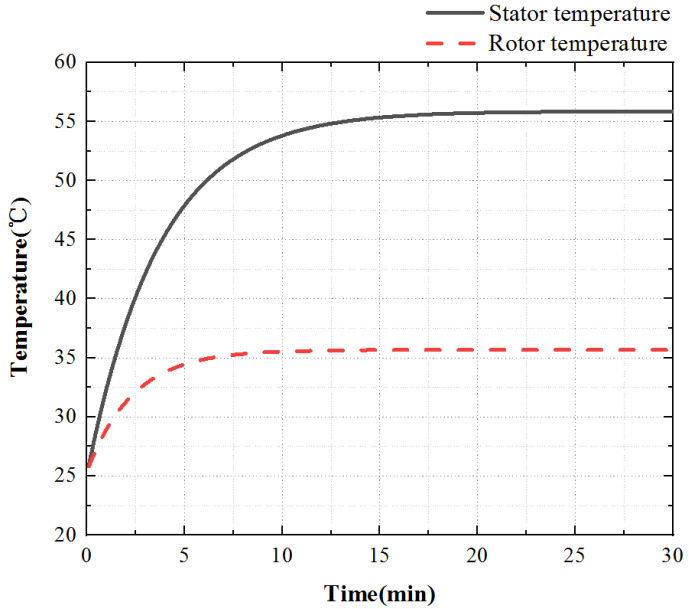
Temperature rise characteristic curve of the contact interface.

Select one point on the rotor and stator teeth at the friction contact interface to obtain the temperature rise curve of these two points, as shown in Figure 19.

In the process of friction heat generation, the rotor surface temperature is always lower than the stator surface temperature and the growth trend of the two is the same, and the rotor temperature reaches a stable state first. When t is 7 min, the rotor reaches the thermal stability state, and when t is 15 min, the stator reaches the thermal stability state.

**Figure 20:**
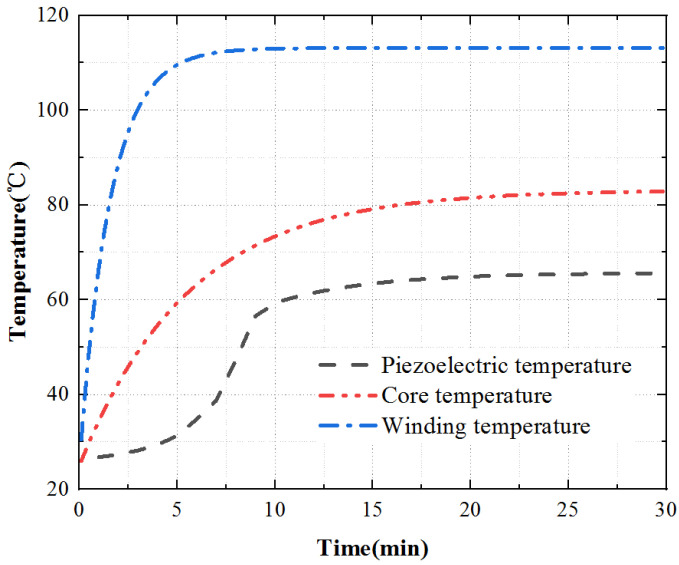
Temperature rise characteristic curve.

To sum up, the temperature rise of the three key points inside the motor is shown in Figure 20. The temperature rise of the stator core is a point on the tooth of the outer stator, the temperature rise of the winding is a point on the winding surface, and the temperature rise of the piezoelectric stator is a point on the tooth of the inner stator surface. The temperature rise of the piezoelectric stator is the coupling iteration of piezoelectric ceramic and contact friction temperature rise. The piezoelectric ceramic sheet is pasted on the stator substrate after silver plating and polarization. Affected by the bonding layer, its temperature rises slowly at first, then quickly, and finally tends to a stable state.

## 5. Experiment

**Figure 21:**
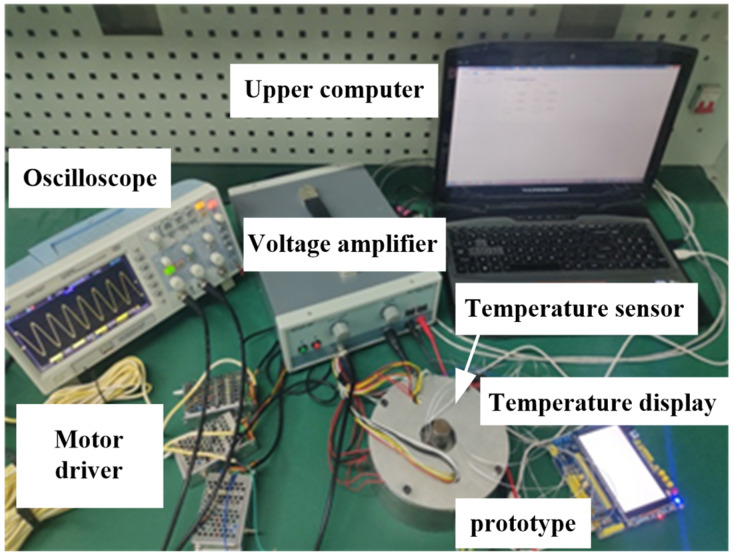
Temperature rise characteristic test platform.

The motor has a special structure of hybrid drive, so the traditional temperature test method of the electromagnetic motor is not applicable. In this paper, the temperature measurement experiment of the temperature sensor based on a micro-thin film thermocouple is designed to detect the temperature rise characteristics of key points in the motor, as shown in Figure 21. When the temperature measurement accuracy is not high, it is considered that the resistance change is linear with the temperature change. The PT100 temperature sensor is stuck to the outer stator tooth, winding surface, and piezoelectric stator tooth edge with an epoxy resin adhesive to gauge the temperature rise of key parts of the motor.

**Figure 22:**
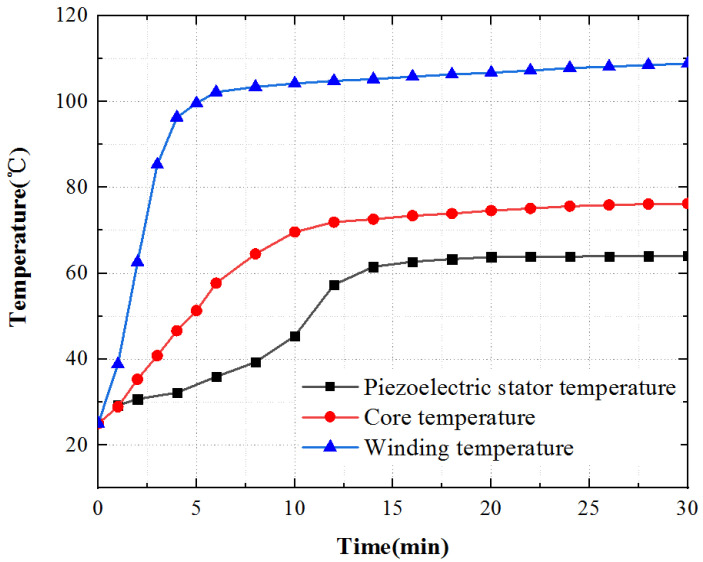
Temperature time characteristic curve.

Apply an excitation signal to the motor, apply three-phase AC with the voltage of 380 V to the electromagnetic part, and apply sine and cosine response inspire with the voltage of 200 V and frequency of 40.575 kHz to the piezoelectric part. Due to the symmetry of the motor structure, select one point on the outer stator tooth, winding surface, and piezoelectric stator tooth, and measure it through a PT100 temperature sensor, and the temperature rise curve of each point is obtained, as shown in Figure 22.

As shown in Figure 22, the motor temperature generally increases rapidly at first, then slowly, and ultimately gradually reaches to be stable. The surface temperature of the motor winding raises the fastest and reaches the stable state first. The temperature increases rapidly in 0~4 min and slowly in 4~6 min. After 6min, the temperature tends to be stable. The surface temperature of the iron core increases more slowly than that of winding, and the temperature tends to be stable after 14 min. The surface temperature of the piezoelectric stator increases, the temperature increases slowly from 0 to 6 min, the temperature increases rapidly from 6 to 14 min, and gradually tends to be stable after 14 min.

**Figure 23:**
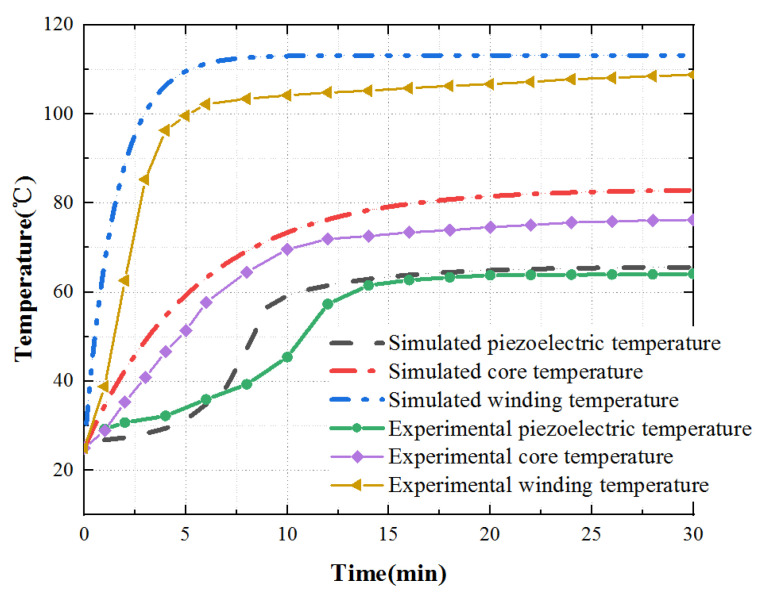
Comparison of simulation and experimental data.

Finally, according to the theoretical analysis, the calculation results are contrasted with the experimental measurement data, as shown in Figure 23.

According to the experimental and theoretical analysis, outcomes show that the temperature rise trend obtained by the experimental and theoretical analysis is consistent. In the steady-state, the experimentally measured value is slightly lower than the theoretical value. This difference is mainly due to the influence of the external environment on the actual situation. In the temperature rise stage, the temperature rise of key parts first increases and then tends to be stable. However, there is a slight difference in the temperature rise of the piezoelectric stator. This difference is because the PCS is stuck to the stator substrate after silver plating and polarization, and the bonding layer will affect the heat transfer. At the same time, due to the influence of the external environment, the temperature rises slowly.

In short, the temperature rise trend obtained from the experimental and theoretical analysis is showing no difference from the expectation. It provides analysis and test methods for further research on heat source optimization and provides a reference for improving motor performance.

## 6. Conclusions

As a new type of hybrid drive motor, combined with the characteristics of electromagnetic drive and piezoelectric drive, EPHDM has important research value. This paper keynotes the serious trouble of the temperature rise of the hybrid drive motors. On the model of the EPHDM, this paper completely analyzes the special structure and working principle of the motor, then establishes a theoretical pattern to analyze the loss, simulates and calculates the temperature rise characteristics, and verifies the temperature rise of the motor through experiments. The calculation outcome makes known that the motor temperature distribution is reasonable, the winding temperature is relatively high, and the iron core temperature and piezoelectric stator temperature are relatively low.

Through the research and analysis, the temperature isoline inside the motor and the temperature rise characteristics of each part are preliminarily understood. In the next step, we will deeply study how to adjust the motor parameters to decrease the temperature rise without affecting the output characteristics of the motor.

## Figures and Tables

**Table 1:** Electromagnetic loss.

Loss	Numerical Value (W)
Stator core loss	23.8
Copper consumption of stator winding	31.1
Eddy’s current loss of permanent magnet	0.026
Rotor eddy’s current loss	0.0073
Mechanical loss	0.018
Total loss	54.9513

**Table 2:** Loss of permanent magnet synchronous motor.

Loss	Numerical Value (W)
Stator core loss	112.83
Copper consumption of stator winding	289.7
Eddy’s current loss of permanent magnet	1.43
Rotor core loss	4.61
Mechanical loss	32.6
Total loss	441.17

**Table 3:** Piezoelectric loss.

Loss	Numerical Value (W)
Vibration loss of PCS	11.9
Stator deformation loss	1.31
Friction loss	16.68
Total loss	29.89

**Table 4:** Loss of ultrasonic motor.

Loss	Numerical Value (W)
Vibration loss of PCS	2.412
Stator deformation loss	3.554
Friction loss	6.264
Total loss	12.23

**Table 5:** Material parameters.

	Attribute	Density (kg/m^3^)	Thermal Conductivity (W/(m∗K))	Specific Heat Capacity (J/(kg∗K))
Material Science				
DW310-35		7600	23	448
Copper		8933	400	385
Phosphor bronze		8800	10	1507.3
PZT-8		7600	5	420
Teflon		2100	0.23	1046

## Data Availability

Not applicable.
